# Eserine and Pilocarpine in the Treatment of Eye Disease

**Published:** 1878-04

**Authors:** 


Eserine and Pilocarpine in the Treatment of Eye Disease.—It is now about fifteen years since the calabar bean (*Physostigma venonosum*) was introduced to the profession as an agent having the till then unattainable quality of producing, at will, contraction of the pupil. Its great value was at once recognized by ophthalmologists. But the supply of the remedy, previously unknown to commerce, was limited, and it is only recently that its alkaloids, eserine and physostigmine, have been readily obtainable for therapeutical purposes and physiological experiment.

During the past two years I have made extensive use of eserine in the treatment of corneal ulcers. The great number of cases of ulceration in strumous children and of traumatic and other ulcerations in adults presenting themselves at the ophthalmic department of the Boston City Hospital, together with those occurring in private practice, have afforded abundant opportunities for observation and comparison, and have allowed of an estimate as to the value of treatment which could not be conclusively based on merely a few cases of a disease so variable in its severity and duration.

The modern treatment of ulceration of the cornea as occurring in young children, which had to a great extent superseded the use of caustics, insufflations of calomel, and counter-irritations, has consisted largely in local applications of solutions of atropia. This has been employed to prevent the occurrence of hernia of the iris in case of corneal perforation, and was also and principally used on the theory that it acted as a sedative upon the affected part. As to this sedative influence, I have long been skeptical, and unless this can be admitted as an undoubted fact, strong objections exist to the indiscriminate use of atropia. By causing a wide expansion of the pupil, and admitting a strong glare of light to the retina, it increases the already intense photophobia, and, by thus exciting still further spasmodic contractions, it tends to keep up the morbid processes by the friction and close pressure of the lids upon the ulcerated surface of the cornea, the very thing it is most important to avoid.

It seemed that eserine, by its strong contractile action on the pupil, limiting very much the amount of light which would reach the retina, might lessen the reflex action causing these spasmodic contractions, and thus prove of great advantage. The results of trial have fully justified my anticipations.

In strumous corneal ulceration in children, there is little chance that the iris will be involved by contiguity; therefore no objection exists to the use of eserine, so far as any fear might be entertained of closing the pupil by effused lymph, except where perforation of the cornea has occurred or is imminent. Even then, if the ulcer is at the margin of the cornea, eserine would be indicated, as it would draw the iris away from the perforation and lessen the danger of hernia iridis. If the ulceration is central, eserine may still be used as a curative means, being replaced at any moment by atropia, if desirable, in case perforation is threatened.

Children of tender age can give little direct information as to their sensations, but, judging from their actions and the repeated testimony of intelligent adults, there is no doubt that a sedative effect, often at least, follows the application of the eserine solution; the supra-orbital pain, which is sometimes one of the physiological sequelæ of its use in a healthy eye, not being felt, but on the contrary a sense of relief from the pain already present in this region.

If we put into the eye a drop of a solution of sulphate of eserine, (two grains to an ounce of water) it causes the pupil to contract strongly in about fifteen minutes, and this effect continues for some eight hours. It should be used in the morning, at which time the photophobia is greatest, so that its effect may continue during the day, and be repeated in the afternoon, if required. Its application causes little or no pain. A solution of eight or ten grains of borax to an ounce of water may also be used twice a day, or oftener, as an auxiliary, to lubricate the ulcerated surface and soothe its irritability.

In phlyctenular or herpetic eruptions of the conjunctiva or of the epithelial layer of the cornea, eserine is of service, especially when photophobia is present, and is far preferable to atropia, which, by causing intolerance of light, adds to the patient's discomfort, and which, also, by exciting spasmodic friction of the lids over the phlyctenular elevations, increases the annoying sensations of a foreign body in the eye. There is, unfortunately, a disposition of late, among general practitioners, to employ atropia as a universal remedy in eye affections, probably because so much has been said of its value in iritis.

In traumatic or gonorrhœal ulceration, in ulceration of the cornea in persons advanced in life, or following exhaustive disease, and in creeping ulcer, (ulcus serpens) my experience with eserine has been favorable. The circum or supra-orbital pain, so often accompanying these ulcers, has been relieved in a marked degree as soon as the remedy had time to act, and the ulceration has assumed a healthier aspect.

I have not yet had an opportunity to employ eserine in the rare but dangerous form of ulcer accompanying some cases of herpes zoster frontalis, but the loss of accommodation and dilatation of the pupil attending this disease, would afford especial indications for its use.

In the paralysis of accommodation and mydriasis often resulting from diphtheria, and sometimes from measles or scarlatina, eserine is very effective in abbreviating the duration of the abnormal condition. In cases of paralysis of the ciliary branch of the third pair, resulting from exposure to the cold, it is similarly useful. In paralysis of this nerve from traumatic or other causes, it is sometimes curative, sometimes only palliative; but even when only the latter, its application, once every day or two, affords much relief in lessening the amount of light, or, in other cases, by reducing the size of the widely-dilated pupil, gives much satisfaction to the patient from its cosmetic effect. In the hysterical photophobia, which sometimes causes seclusion from light, even for years, eserine forms an important part of the treatment.

Having observed a lessening of previously existing injection of the ciliary region after its application, (a fact which seems to me important), I should hope for advantage from its use in the commencement of sympathetic irritation of one eye after traumatic injury of the other; but it should be used only as a means of arresting the morbid process after proper measures have been taken for the removal of the source of sympathetic mischief. It, as well as pilocarpine, may be similarly useful in episcleritis. In an instance of extremely conical cornea, I have surprised and delighted the patient by the great improvement in vision obtained by the use of eserine.

The obvious effects of the installation of a drop of a solution of two grains of eserine sulphate in an ounce of water into a healthy eye, usually begin to manifest themselves within fifteen minutes. The pupil contracts strongly, becoming, perhaps, not more than a millimetre in diameter; there is often twitching of the lids, and sometimes supra-orbital pain, which, usually slight, may be considerable. Vision is dim, as if the sun were eclipsed. This dimness depends on the narrowness of the pupil, which admits of the passage of only a limited amount of light. There is also spasm of the accommodation, and an induced myopia, which often reaches in a few minutes a very high degree. If the latter is corrected by a concave glass of equivalent power, vision for large objects becomes nearly normal.

These symptoms are usually at their height within an hour, after which they diminish, and at the end of the second hour have in most cases disappeared, with the exception of the contraction of the pupil, which persists for perhaps eight hours or longer.

The above facts are results of my own clinical observation. In the last and the preceding number of Graefe's *Archiv fuur Ophthalmologie*, Vol. xxiii., Parts II. and III., just received, as also in Vol, xxii., No. 4, I find accounts of careful and elaborate experiments and observations made by Drs. A. Weber and Mohr, of Darmstadt, Von Reuss, of Vienna, and Professor de Laqueur, of Strasburg, regarding the action of eserine upon healthy and diseased eyes. These have great value as explaining the *modus operandi*

of this medicament, and as affording ground for the belief that it is to prove of extended application in ocular therapeutics, and they confirm in all respects the conclusions I had arrived at.

As regards the effects of eserine upon the cornea, the researches of these gentlemen seem to prove that the activity of the circulation is increased, that the pressure within the anterior chamber is lessened, that the action of accommodation is excited, and that the radius of curvature is shortened during its use. Increased activity in the blood supply, by rendering the cornea more highly vitalized, favors the removal of effete particles and the establishment of a process of repair; the diminished pressure upon the cornea (this pressure being itself a potent cause of ulceration) tends to limit the depth of the ulcer, and lessens the danger of perforation. Dr. von Wecker, of Paris, also believes that the eserine prevents the pus from being reproduced in cases of corneal abscess, and in suppuration after cataract operation. We have thus a rational explanation of the benefit derived from the use of eserine in corneal affections.

Dr. Weber considers that the indications for the therapeutic use of extract of calabar bean and its still more efficient alkaloid may be at once deduced from a knowledge of its physiological and, as we may say, mechanical effects. Following these indications in a great number of corneal affections he gives the results, which I translate from his own words:

"Calabar has its greatest triumph and its widest application in deep corneal ulceration, and we can assert that the therapeutic value of the means usually employed, such as compressive bandages, warm fomentations, paracentesis, iridectomy, etc., is, with few exceptions, insignificant in comparison with the great efficacy of calabar.

"It appears clearly, from my experiments, that atropine, which is used so generally, and, as I may say, in such a slap-dash manner (*schablonenhaft*), in these affections, increases the infra-corneal pressure to a dangerous degree, and hastens perforation of the corneal ulcer."

Drs. Weber and Laqueur commend the use of eserine, as also of pilocarpine, in glaucoma, not at present, at least, as a substitute for the operative treatment by iridectomy, but as auxiliary means. In their opinion these remedies may arrest the symptoms at the premonitory stage by lessening the intra-ocular tension and relieving the obstructed circulation, and may also prevent a threatened relapse, indicated by a renewal of abnormal tension, after an attack for which iridectomy had been successfully performed.

At the meeting of the Heidelberg Ophthalmologische Gesellschaft in September, 1875, Dr. von Wecker spoke of pilocarpine, the alkaloid of jaborandi, as a myotic, and at the Societe de Biologie st Paris, October, 1877, Dr. Galezowski stated that he had found the nitrate of pilocarpine, which caused no irritation when applied to the conjunctiva, equally as effective as eserine. His experience was confirmed by Dr. Galippe.

In my own experiments, made with the chlor-hydrate of pilocarpine, the results obtained have differed a little from those produced by eserine sulphate, in the facts that less conjunctival irritation, less supra-orbital pain, and less spasm of the accommodative power seemed to be induced, while the contraction of the pupil and the temporary myopia corresponded in degree with those following the use of eserine. In these respects pilocarpine offers great advantages over eserine. It is, moreover, at present less costly than eserine, and it does not, as does the latter, deliquesce on keeping.

We have, therefore, unquestionably, two myotic agents capable of rendering immense service in ocular affections, and probably of use in other diseases, especially of the nervous system.

It is needless to say that these, as all other remedies, have their limitations of usefulness; in iritis, for instance, eserine and pilocarpine would doubtless be highly injurious, as tending to congest the already distended vessels, and as favoring the formation of adhesions between the iris and the capsule of the crystalline lens.

